# Antioxidant and Anti-Inflammatory Activities of Phytoecdysteroids from *Vitex madiensis* (Oliv.)

**DOI:** 10.3390/molecules31122110

**Published:** 2026-06-15

**Authors:** Ghislaine Boungou-Tsona, Caroline Decombat, Kevin Bikindou, Maël Gainche, Isabelle Ripoche, Laetitia Delort, Florence Caldefie-Chézet, Aubin Nestor Loumouamou, Pierre Chalard

**Affiliations:** 1 Institut National de Recherche en Sciences Exactes et Naturelles (IRSEN), Cité Scientifique, Brazzaville BP 2400, Congo; ghislaine.boungou-tsona@univ-smb.fr (G.B.-T.);; 2 ICCF, Clermont Auvergne INP, Université Clermont Auvergne, CNRS, F-63000 Clermont-Ferrand, France; mael.gainche@sigma-clermont.fr (M.G.);; 3 Equipe Pluridisciplinaire de Recherche en Alimentation et Nutrition (EPRAN), Faculté des Sciences et Techniques, Université Marien Ngouabi, Brazzaville BP 389, Congo; 4 UNH, Unité de Nutrition Humaine, CRNH Auvergne, INRAE, Université Clermont-Auvergne, F-63000 Clermont-Ferrand, France; 5 Laboratoire de Chimie des Extraits Végétaux, Ecole Normale Supérieure, Université Marien Ngouabi, Brazzaville BP 69, Congo

**Keywords:** *Vitex madiensis*, antioxidant, antiinflammatory, phenolic compounds, phytoecdysteroids

## Abstract

*Vitex madiensis* Oliv. (*Lamiaceae*) is a species growing in tropical and subtropical regions throughout the world. In several African countries, the different organs of this plant, leaves, fruits, stem bark and roots are used in folk medicine for the treatment of headaches, toothaches, aches and pains. In this study, we investigated the phytochemical profile of *Vitex madiensis* leaf extracts using LC-MS. The antioxidant and anti-inflammatory potential of crude extracts, fractions, and pure molecules was evaluated using reactive oxygen species (ROSs) production assays and cyclooxygenase-2 inhibition assays. A bio-guided fractionation was carried out to identify the most active fractions and resulted in the isolation of four phytoecdysteroids from the n-butanol fraction: 20-hydroxyecdysone, ajugasterone C, vitexirone, and pterosterone. 20-hydroxyecdysone showed very good anti-inflammatory properties with a significant reduction of more than 70% of *COX-2* expression in induced LPS-stimulated human blood leukocytes compared to the control. This study confirmed the therapeutic potential of phytoecdysteroids.

## 1. Introduction

The genus *Vitex* is composed of around 270 identified species, most of which grow in tropical regions around the world. In Africa, 110 species have been identified and many of them are part of the therapeutic arsenal of traditional African medicine. *Vitex madiensis* appears as a shrub that can reach 5 m high, with a massive underground woody rootstock. The different parts (leaves, fruits, stem bark and roots) are used for food and medicinal purposes [1,2,3]. Indeed, several authors have described the use of *Vitex madiensis* extracts in the traditional treatment of fever, malaria, insomnia, pain and inflammation [4,5,6,7]. Other studies have highlighted antihelminthic [8], hypoglycemic, anti-hyperglycemic and antibacterial activities of *Vitex madiensis* extracts [9,10,11,12,13]. If the chemical profile of some species is well described, such as *Vitex negundo* L., *Vitex rotundifolia* L. or *Vitex agnus castus* L., the chemical composition of *Vitex madiensis* is not well known. Studies on *V. madiensis* demonstrate the presence of flavonoids, iridoids, phenolic acids and derivatives, and phytoecdysteroids [14,15,16,17]. Ecdysteroids belong to the steroid hormone family, which were first isolated from arthropods and play a crucial role in the molting process, development, and reproduction. In plants, this family of compounds is more abundant and is involved in the plant’s defense mechanism against invertebrate predation. Since they were discovered, a lot of studies demonstrate their biological and therapeutic activities [18,19,20,21,22,23,24]. Indeed, phytoecdysteroids are described to possess a wide range of therapeutic activities such as antidiabetic, anti-stress, spasmolytic and anti-inflammatory activities. They have also been studied for their ability to decrease the formation of amyloid plaques, present in the brain of Alzheimer’s patients [25].

The phytochemical profile of four parts of *V. madiensis* (leaves, trunk barks, root barks, and fruits) has been previously described [17]. It was observed that each part contains phytoecdysteroids such as 20-hydroxyecdysone, vitexirone, and ajugasterone C. The methanolic extract of the leaves exhibited both the best free radical scavenging activity (DPPH) and the greatest inhibition of reactive oxygen species (ROS) production by leukocytes. Therefore, to investigate the anti-inflammatory potential of this methanolic extract of *V. madiensis* leaves, bioguided fractionation was performed in order to determine which metabolites are responsible for such activities and to evaluate their anti-inflammatory activity by exploring their effects on COX-2 expression in lipopolysaccharide (LPS)-stimulated human blood leukocytes.

## 2. Results

### 2.1. Bioguided Fractionation of the Methanolic Extract of Vitex madiensis Leaves

It was previously reported the phytochemical profile of the methanolic extract of *V. madiensis* leaves carried out by LC-MS analysis. Several families of metabolites such as flavonoids (vitexin, homoorientin, orientin, vicenin, luteolin), iridoids (1-oxo-eucommiol, agnuside), organic acids (quinic acid, citric acid, dicaffeoquinic acid derivatives), and phytoecdysteroids, considered as taxonomic markers of the *Vitex* genus [26]. We supposed the presence of five specific phytoecdysteroids: 20-hydroxyecdysone, ajugasterone C, vitexirone, isovitexirone and pterosterone.

**Figure 1:**
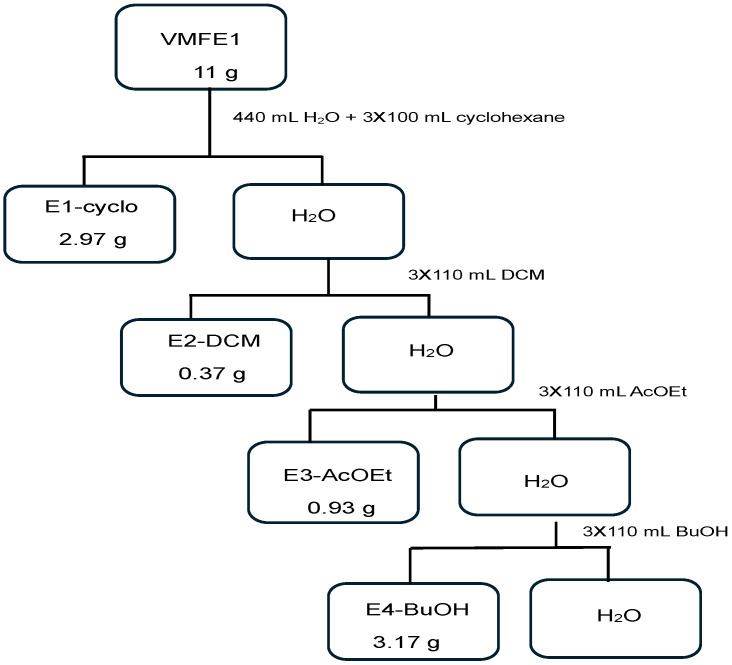
Bioguided fractionation of methanolic crude leaves extract.

To validate the structure of the five phytoecdysteroids of *V. madiensis* and evaluate their potential anti-inflammatory activity, bioguided fractionation was performed using four different organic solvents of increasing polarity: cyclohexane, methylene chloride, ethyl acetate and butanol. Five fractions were obtained: E1-cyclo (cyclohexane, 27% yield), E2-DCM (methylene chloride, 3.5% yield), E3-AcOEt (ethyl acetate, 8.5% yield), E4-BuOH (butanol, 28% yield) and E5-H_2_O (water, 27% yield) which were analyzed both by LC/MS to determine the chemical composition and to evaluate their ability to reduce ROS production (Figure 1).

### 2.2. Phytochemical Profiles

The analyses were performed using the LC method previously described to characterize the methanolic extract profile [17]. Analysis showed that the fractions E1-cyclo and E2-DCM contained apolar compounds such as organic acids (octadecenedioic acid) and flavonoids (dimethyl quercetin and luteolin) (Table 1). The five potential ecdysteroids, 20-hydroxyecdysone, ajugasterone C, vitexirone, isovotexirone and pterosterone were mostly present in the fractions E3-AcOEt and E4-BuOH. These fractions were also composed of glycosylated flavonoids (luteolin-4′-O-glucoside, orientin) and caffeoylquinic derivatives. E5-H_2_O contained mainly sugars, organic acids (citric acid and quinic acid) and a small amount of ecdysteroids.

### 2.3. Antioxidant and Anti-Inflammatory Activities

**Figure 2:**
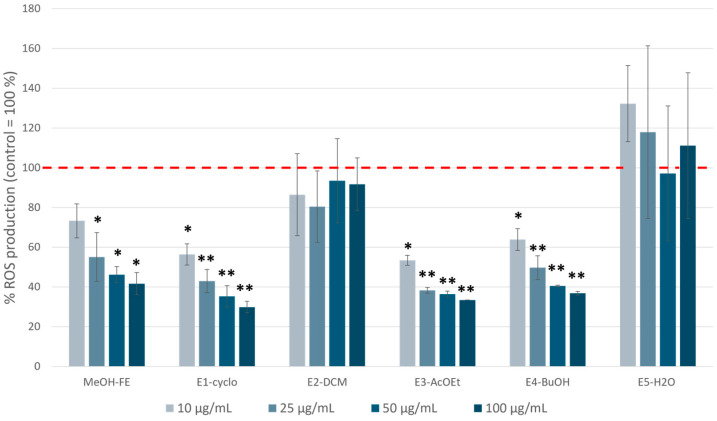
ROS production of blood leukocytes incubated with the methanolic extract of *V. madiensis* leaves (MeOH-FE) or fractions (10, 25, 50 and 100 μg/mL) and stimulated with PMA (1 μM) for 2 h. Values are expressed as percentage of the Control (cells incubated with PMA and without extract). Data are shown as means ± SEM (n = 3–4); * *p* < 0.05, ** *p* < 0.01 compared with Control normalized as 100% (red dashed line).

A range of concentrations was evaluated for each fraction (0 [control], 10, 25, 50, and 100 µg/mL). The observed effects were subsequently compared with those obtained for the crude methanolic extract of *V. madiensis* leaves tested at identical concentrations (Figure 2).

Incubation with the crude methanolic extract significantly inhibited ROS production from a concentration of 25 µg/mL (around 45% inhibition). After 2 h, a significant reduction of minimum 50% was obtained at 50 µg/mL and above. The E1-cyclo, E3-AcOEt, and E4-BuOH fractions also significantly decreased ROS production. A reduction of more than 50% at 25 µg/mL and above was noted (−57%, −62% and −50% for E1-cyclo, E3-AcOEt and E4-BuOH, respectively). The fraction with the best activity was E3-AcOEt, with 47% inhibition from the lowest concentration (10 µg/mL). The E1-cyclo and E4-BuOH fractions, with 44% and 37% inhibition respectively, also appeared to have interesting activity.

Our results are consistent with numerous studies reporting the antioxidant activity of plant-derived extracts. Recent investigations have shown that extracts from *Origanum vulgare* and leaves of *Fragaria* × *ananassa* significantly reduce intracellular reactive oxygen species (ROS) levels, an effect that has been attributed to their high polyphenol content [39]. In addition, methanolic extracts prepared from both the aerial parts and roots of *Scutellaria salviifolia* Benth. (Lamiaceae) have demonstrated a strong antioxidant capacity, together with a marked inhibition of cyclooxygenase2 (COX-2), reaching levels comparable to those of the selective COX-2 inhibitor celecoxib [40]. Phytochemical analyses of these extracts revealed a high abundance of flavonoids and phenolic acids, notably derivatives of luteolin, apigenin, and quercetin, compounds well known for their combined antioxidant and antiinflammatory properties. Taken together, these findings indicate that many plant extracts exert dual antioxidant and antiinflammatory activities, acting both as scavengers of reactive oxygen species and as inhibitors of inflammatory enzymes, such as COX-2 [41]. On this basis, the antiinflammatory potential of compounds present in the leaf extracts of *V. madiensis* were evaluated, with particular emphasis on the isolation of ecdysteroids from the butanolic fraction (E4-BuOH), which was the most abundant fraction and exhibited one of the strongest antioxidant activities.

### 2.4. Isolation of Phytoecdysteroids and Antioxidant Activity

#### 2.4.1. Isolation and Identification of Phytoecdysteroids in E4-BuOH Fraction

**Figure 3:**
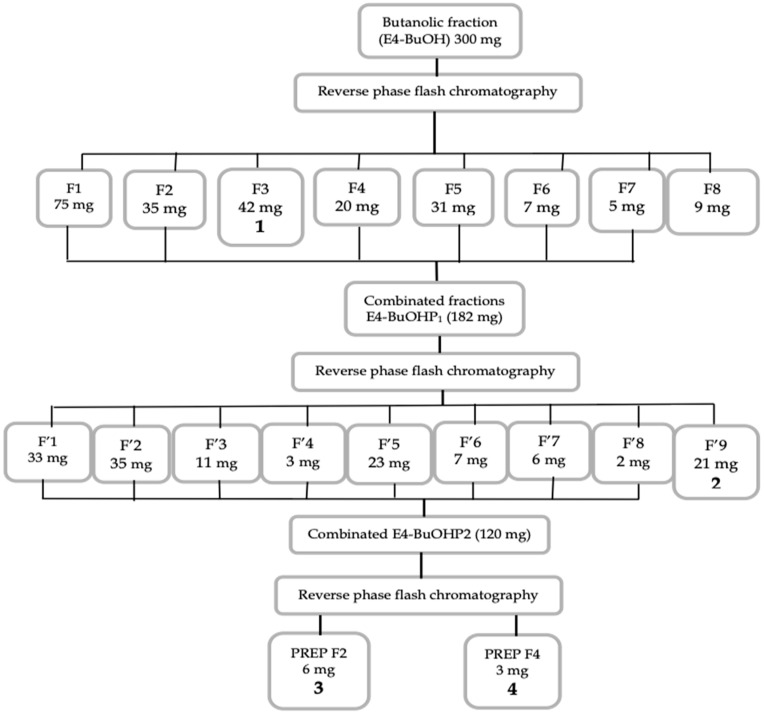
Isolation of compounds **1**, **2**, **3** and **4** from butanolic extract.

The butanol fraction was first purified by reverse phase flash chromatography to afford eight subfractions (Figure 3).

**Figure 4:**
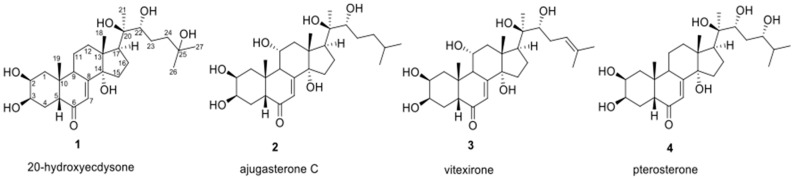
Ecdsysteroids isolated from butanolic fraction.

One of these fractions contained a pure compound which was identified as 20-hydroxyecdysone **1** by NMR (Table 2) and HRESIMS analyses ([M+H]^+^ for C_27_H_44_O_7_, *m*/*z* 481.3152 (calculated 481.3159) [34,42]. The other subfractions were combined and purified by reverse-phase flash chromatography. This purification allowed us to isolate ajugasterone C **2**. Its structure was confirmed by NMR and HRESIMS analyses ([M+H]^+^ at *m*/*z* 481.3152 (calculated 481.3159)). The other fractions resulting from this purification were engaged in a preparative HPLC reverse phase to obtain the last two ecdysteroids: vitexirone **3** and pterosterone **4** (Figure 4).

The structures of vitexirone and pterosterone were determined by NMR and HRESIMS analyses (vitexirone: [M + H]^+^ at *m*/*z* 479.2998 (calculated: 479.30033); pterosterone: [M + H]^+^ at *m*/*z* 481.3152 (calculated: 481.3159)). The ^1^H and ^13^C NMR data presented in Table 2 are consistent with those obtained by other authors [43,44,45], thus confirming the structures of these four isolated phytoecdysteroids.

#### 2.4.2. Antioxidant Activity of Isolated Phytoecdysteroids

**Figure 5:**
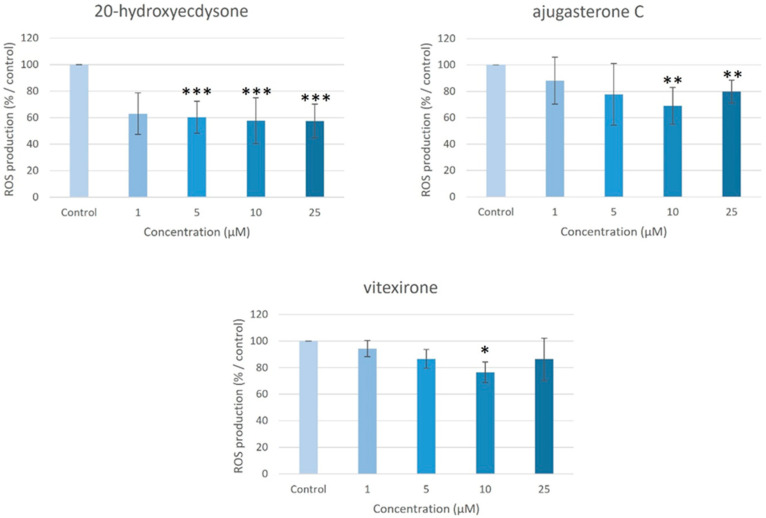
ROS production of blood leukocytes, incubated with three of the isolated ecdysteroids compounds (20-hydroxyecdysone **1**, ajugasterone C **2**, vitexirone **3**) from *Vitex madiensis* leaves (1, 5, 10, 25 µM) and stimulated with PMA (1 μM) for 2 h. Values are expressed as percentage of the Control (cells incubated with PMA and without extract). Data are shown as means ± SEM (n = 3–6); * *p* < 0.05, ** *p* < 0.01, *** *p* < 0.001 compared with Control normalized as 100%.

20-hydroxyecdysone **1**, ajugasterone C **2** and vitexirone **3**, isolated from the E4-BuOH fraction, were tested on the production of ROS by human stimulated blood leukocytes according to the same protocol as described above at different concentration (0 (Control), 1, 5, 10, and 25 µM). Pterosetrone **4** could not be tested due to the small amount of product collected. The results showed a significant decrease in ROS production by total leukocytes after 2 h of incubation in the presence of ajugasterone C (−31% at 10 µM, −20% at 25 µM, *p* < 0.01), and vitexirone (−24% at 10 µM, *p* < 0.05) (Figure 5). The most effective compound is 20-hydroxyecdysone, which reduced ROS production by at least 37% from the concentration of 1 µM.

**Figure 6:**
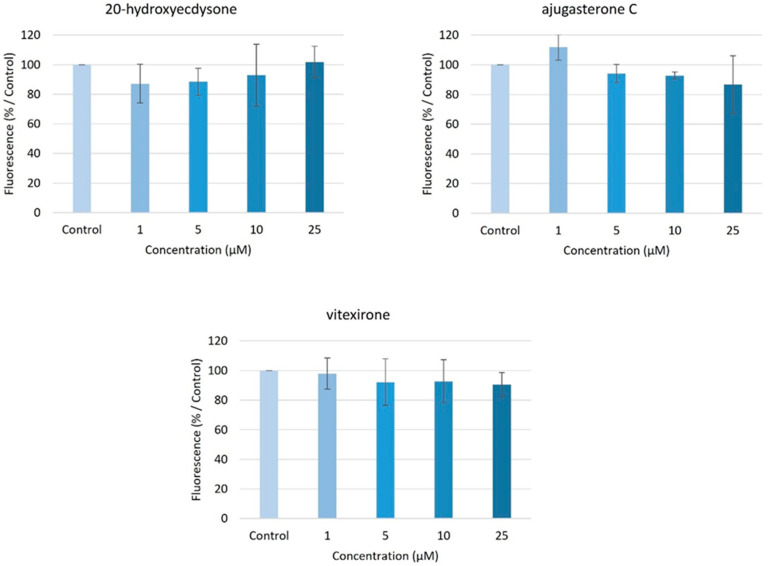
Effect of phytoecdysteroids on leukocyte viability. Cells were treated with the indicated concentrations for 2 h, and cell viability was measured. Data were shown as means ± SEM (Control = 100%) (n = 3–6).

This observed effect cannot be attributed to alterations in cell viability or proliferation, since the resazurin-based viability assay revealed no statistically significant differences between cells treated or not with the various pure compounds at concentrations ranging from 1 to 25 µM after 2 h of incubation (Figure 6).

### 2.5. Anti-Inflammatory Potential of 20-Hydroxyecdysone

**Figure 7:**
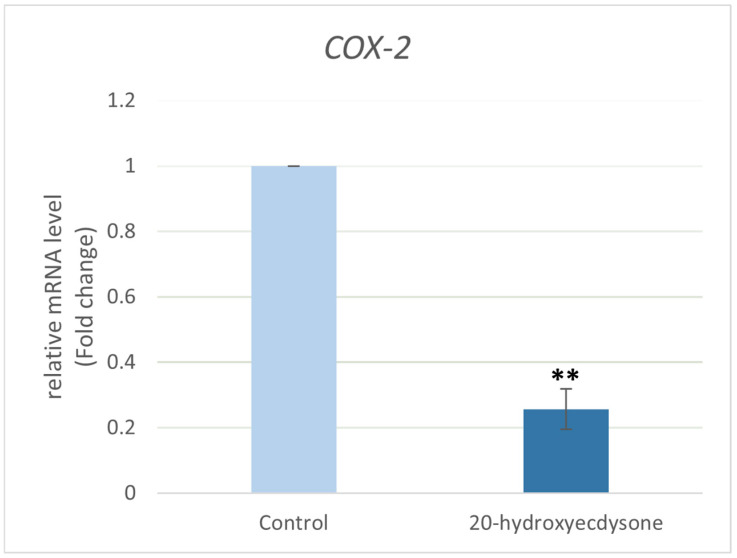
Impact of 20-hydroxyecdysone (1 µM) on the relative expression of *COX-2* in leukocytes incubated 4 h with LPS (10 µg/mL). The results measured by qPCR were expressed as percentage of expression compared to the Control (leukocytes stimulated by LPS, without 20-hydroxyecdysone) set at 100%. **: *p* < 0.01. Three independent experiments were performed.

As 20-hydroxyecdysone gave better results in terms of antioxidant activity, its anti-inflammatory potential was assessed. To this end, its effect on the inflammatory response was studied by focusing on the COX-2 pathway. The cyclooxygenase-2 enzyme is expressed in response to inflammatory stimuli and leads to the production of prostaglandins, products contributing to the inflammatory response among others. The expression of the *COX-2* gene by total leukocytes stimulated by LPS (1 µg/mL) for 4 h was measured in the presence of 20-hydroxyecdysone (1 µM). 20-hydroxyecdysone significantly reduced the relative expression of the *COX-2* gene by more than 70% (*p* ≤ 0.01) compared to the Control (LPS-stimulated blood leukocytes) (Figure 7). This result confirmed the anti-inflammatory potential of this molecule.

The marked reduction in *COX-2* gene expression induced by 20-hydroxyecdysone strongly suggests an involvement of the arachidonic acid pathway in its biological activity. As COX-2 is a key enzyme in the synthesis of pro-inflammatory prostaglandins, its downregulation may partially account for the observed effects. Nevertheless, the precise molecular targets and downstream signaling events remain to be elucidated. Further studies assessing the expression and activity of key enzymes involved in the arachidonic acid cascade, including upstream phospholipases and downstream prostaglandin synthases, as well as post-transcriptional regulation and prostaglandin production, would be required to better delineate its precise mechanism of action.

It has been reported in the literature that ajugasterone C has anti-inflammatory effects through the inhibition of agar-induced paw edema in rats and 20-hydroxyecdysone exhibits antioxidant and anti-inflammatory effects on several cell lines [14,15,18,46]. In the context of the COVID-19 pandemic, 20-hydroxyecdysone was part of the therapeutic arsenal to try to treat certain pathologies which impacted the respiratory function of people affected by this disease. Indeed, the anti-inflammatory, antithrombotic and antifibrotic properties of 20-hydroxyecdysone have been exploited to develop a treatment likely to improve the respiratory function of patients developing severe forms of pneumonia [47]. Our results showed that 20-hydroxyecdysone caused a significant reduction in the relative expression of COX-2 induced by immune cells, thus confirming its anti-inflammatory potential.

## 3. Materials and Methods

### 3.1. Plant Material

The leaves of *Vitex madiensis* were collected in the savannah zone, 25 km South of Brazzaville. After collection of the samples, one specimen was authenticated and preserved in the herbarium of the National Institute for Research in Exact and Natural Sciences (IRSEN) of Brazzaville under the number T7041. The plant material was dried in the shade for 7 days and then ground with a Biomix type grinder (Biolomix, Dongguan, China). Thus the resulting powder was stored in a plastic bag prior to extraction.

### 3.2. Cell Material

Blood was collected from healthy human volunteers (n = 6; French Blood Establishment, EFS, Clermont-Ferrand, France). Donors gave their written informed consent for the use of blood samples for research purposes under EFS contract n° 16-21-62 (in accordance with the following articles L1222-1, L1222-8, L1243-4 and R1243-61 of the French Public Health Code). Whole blood leukocytes were obtained as described in the literature [48,49]. Briefly, leukocytes were obtained by hemolytic shock (NH_4_Cl 155 μM; NaHCO_3_ 12 μM, EDTA 0.01 μM). Leukocytes (10^6^/mL) were then suspended in Roswell Park Memorial Institute medium-1640 (RPMI-1640, Gibco, Thermo Fisher Scientific, Waltham, MA, USA) supplemented with fetal bovin serum (FBS, 10%) (Eurobio Scientific, Les Ulis, France), gentamicin (50 μg/mL), and glutamine (Gln, 2 mM) (Thermo Fisher Scientific).

### 3.3. Methanolic Extract and Fractionations

The powder obtained after grinding the plant material was macerated in methanol for 24 h (plant/solvent ratio: 1/10). The mixture was then filtered and evaporated under vacuum to dryness to obtain the methanolic crude extract. The obtained crude extract was run through a liquid/liquid fractionation process successively, with cyclohexane, dichloromethane (DCM), ethyl acetate (EtOAc), n-butanol (n-BuOH) and water (H_2_O). The crude extract and the five fractions E1-cyclo, E2-DCM, E3-AcOEt, E4-BuOH and E5-H_2_O were stored at 4 °C before analysis.

The most active E4-BuOH fraction (300 mg) was then fractionated by flash chromatography using a C18 column and a water–acetonitrile eluent (80:20, *v*:*v*) to obtain eight fractions (1–8). The fraction F3 contained 20-hydroxyecdysone (42 mg). All other fractions were pooled and engaged in another reverse-phase flash chromatography using the same eluent to obtain nine fractions. Among them, fraction F’9 was constituted of pure ajugasterone C (21 mg). The remaining fractions were mixed to form a single fraction (120 mg) which was purified by semi-preparative chromatography in isocratic mode using a water–acetonitrile mixture (80:20, *v*:*v*) as the eluent to obtain vitexirone (6 mg) and pterosterone (3 mg).

### 3.4. Determination of the Chemical Profiles

The chemical profile of the methanolic extract was determined by chromatographic analyses using Ultra-High Performance Liquid Chromatography (UHPLC). HPLC-MS analyses were performed on an Ultimate 3000 RSLC UHPLC system (Thermo Fisher Scientific Inc., Waltham, MA, USA) coupled to a quaternary rapid separation pump (Ultimate autosampler) and a rapid separation diode array detector. Compounds were separated on an Uptisphere Strategy C18 column (250 × 4.6 mm, 5 µm, Interchim, Montluçon, France), which was controlled at 30 °C. The mobile phase was a mixture of 0.1% (*v*/*v*) formic acid in water (phase A) and 0.1% (*v*/*v*) formic acid in acetonitrile (phase B). The gradient of phase A was 100% (0 min), 80% (10 min), 73% (35 min), 0% (40–50 min) and 100% (51–60 min). The flow rate was 0.8 mL/min, and the injection volume was 5 µL. The UHPLC system was connected to an Orbitrap (Thermo Fisher Scientific Inc., Waltham, MA, USA) mass spectrometer, operated in the positive and negative electrospray ionization mode. Source operating conditions were: 3 kV spray voltage; 320 °C heated capillary temperature; 400 °C auxiliary gas temperature; sheath, sweep and auxiliary gas (nitrogen) flow rate 50, 10 and 2 arbitrary units, respectively; and collision cell voltage between 10 and 50 eV. Full scan data were obtained at a resolution of 70,000, whereas MS^2^ data were obtained at a resolution of 17,500. Data were processed using Xcalibur version 4.6 software (Thermo Fisher Scientific Inc., Waltham, MA, USA). The sample to be analyzed was prepared under the following operating conditions: 5 mg of the methanolic extract was diluted in 5 mL of MeOH of HPLC grade quality. The solution is then filtered with a 0.45 m PTFE filter (Merck, St. Quentin Fallavier, France). Part of the filtrate is placed in a vial for analysis.

### 3.5. Sample Preparation

For experiments with cells, methanolic dry extract or isolated products were dissolved extemporaneously in DMSO and then further diluted (0, 10, 25, 50 and 100 µg/mL or 0, 1, 5, 10, 25 µM respectively) with complete culture medium before use. For control conditions, an equivalent amount of solvent was added in the medium.

### 3.6. ROS Production Inhibition by Blood Leukocytes

The ROS production inhibition was determined using human blood leukocytes. The leukocyte preparations (n = 3–6) were obtained as previously described. The cells were placed in a 96-well polystyrene plate (Cell Wells, Corning^®^, Corning, NY, USA), incubated with various extracts added at different concentrations (0, 10, 25, 50, 100 µg/mL) or purified compound (0, 1, 5, 10, 25 µM) and dihydrorhodamine 123 (DHR, 1 µM, Cayman Chemical Company, Ann Arbor, MI, USA) and finally stimulated or not (to check the stimulation efficiency) by phorbol 12-myristate 13-acetate (PMA, 1  µM, Sigma-Aldrich, Saint Louis, MO, USA), as previously described by Nehme et al. [50]. Fluorescence readings (excitation/emission: 485/535 nm) in kinetics of rhodamine 123, which is the product of DHR 123 oxidation by ROS, were taken over 2 h every 10 min using a Tecan Spark^®^ (Männedorf, Switzerland) apparatus. These experiments were repeated three times and the percentage of ROS production in the presence of the extracts was calculated. ROS production was calculated relative to 100% normalized control (untreated cells).

### 3.7. Leukocyte Viability

The same leukocytes preparations were placed in 96 well polystyrene plates incubated with various purified compounds added at different concentrations (0, 1, 5, 10, 25 µM) and finally stimulated or not by PMA (1 µM). After 2 h of incubation at 37 °C under a 5% CO_2_ atmosphere, resazurin (Sigma-Aldrich) was added to each well at the concentration 25 µg/mL. Leukocytes viability and then cytotoxicity of the different extracts were monitored by fluorescence reading (excitation/emission: 530/590 nm) using a Tecan Spark^®^ (Männedorf, Switzerland) instrument. The percentage of cell viability was calculated relative to 100% normalized control.

### 3.8. Evaluation of Gene Expression by Real-Time Quantitative PCR (RT-qPCR)

Leukocytes (10^6^ cells/mL) (n = 3) were incubated with or without lipopolysaccharide (LPS) (10 μg/mL, LPS O26:B26, Sigma-Aldrich) and 20-hydroxyecdysone (0 or 1 µM) for 4 h.

Following the incubation, Trizol reagent (Invitrogen, Carlsbad, CA, USA, Thermo Fisher Scientific) was used to extract total RNA and 1 μg per sample was treated with DNAse I (Invitrogen, Thermo Fisher Scientific) to remove remaining genomic DNA. The RT was undertaken with the MultiScribe reverse transcriptase (High Capacity cDNA Reverse Transcription Kit, Applied Biosystems, Thermo Fisher Scientific) using the StepOne Real-Time PCR System (Applied Biosystems, Waltham, MA, USA) according to the manufacturer’s recommendations. Amplification reaction assays were carried out using SYBRGreen reagent (Thermo Fisher Scientific).

The analysis was conducted on *COX-2* gene and one reference gene (*GAPDH*) used as internal control for normalization of RNA for leukocytes [*GADPH* (Human, Forward Primer Sequence (5′-3′): 5′-CACATGGCCTCCAAGGAGTAA; Reverse Primer Sequence (5′-3′): 5′-TGAGGGTCTCTCTCTTCCTCTTGT); *COX-2* (Human, Forward Primer Sequence (5′-3′): 5′-CCCAGGGCTCAAACATGATG; Reverse Primer Sequence (5′-3′): 5′-TCGCTTATGATCTGTCTTGAAAAACT)]. The thermal cycling conditions were 50 °C for 2 min followed by an initial denaturation step at 95 °C for 10 min, 40 cycles at 95 °C for 30 s, 60 °C for 30 s and 72 °C for 30 s. The relative quantification method (RQ = 2^−ΔΔCT^) was used to compute the relative gene expression with ΔΔCT = [ΔCT (sample1) − ΔCT (sample2)] and ΔCT = [CT (*COX-2* gene) − CT (*GAPDH* gene)]. Paired *t*-test was used for comparison of gene-expression levels. Three independent experiments were performed.

### 3.9. Data Analysis

Data were expressed as the mean ± SEM and represented at least three independent experiments. Statistical analysis and significance were measured using the paired, bilateral Student’s *T*-test. The *p* values were determined, and values <0.05, <0.01, <0.001 (*, **, ***, respectively) were considered significant.

## 4. Conclusions

The bioguided fractionation of methanolic extract of *Vitex madiensis* leaves revealed the presence of phytoecdysteroids, which are the taxonomic markers of *Vitex* species, in bioactive fractions. Thus we isolated and characterized four phytoecdysteroids from the butanolic fraction: 20-hydroxyecdysone **1**, ajugasterone C **2**, vitexirone **3** and pterosterone **4**. The antioxidant activity of compounds **1**, **2** and **3** was evaluated and results showed a significant decrease in ROS production by leukocytes after 2 h of incubation in the presence of these three compounds. Moreover, 20-hydroxyecdysone exhibited a much greater dose-dependent inhibition. The results obtained are even more interesting since none of these compounds showed a cytotoxic effect on cell viability. The anti-inflammatory potential of 20-hydroxyecdysone was evaluated on *COX-2* gene expression studied by qPCR. This compound showed very good anti-inflammatory potential with a significant reduction of more than 70% in the relative expression of *COX-2* by cells compared to the control. The results obtained confirmed that phytoecdysteroids have interesting anti-inflammatory properties, which could justify the practices of traditional healers who use *Vitex madiensis* to treat illnesses involving the inflammatory process. However, it would be interesting to refine these results through additional analyses of the activity of 20-hydroxyecdysone on the arachidonic pathway (COX-1 and pro-inflammatory prostaglandins PGE2) or on the pro-inflammatory or anti-inflammatory cytokines secretion and to explore its mechanism of action.

## Figures and Tables

**Table 1:** Phytochemical profile of the five fractions obtained from methanolic extract of *Vitex madiensis* leaves.

N°	Compound	tr (min)	Formula	M-H (*m*/*z*)	M+ (*m*/*z*)	MS^2^ (*m*/*z*)	Cyclo	CH_2_Cl_2_	AcOEt	*n*-BuOH	H_2_O	Reference
1	Glucose	3.25	C_6_H_12_O_6_	179.0555			−	−	−	−	+	Standard
2	Quinic acid	3.96	C_7_H_12_O_6_	191.0553		191/85/127/93/85	−	−	−	−	++	Standard
3	Citric acid	6.91	C_6_H_8_O_7_	191.0189		191/129/11/87/85	−	−	−	−	++	Standard
4	1-oxo-eucommiol	7.72	C_9_H_14_O_5_	201.0758		201/109/139	−	−	−	−	++	[27]
5	5-Ethylidene-2-hydroxy-2-hydroxymethyl-3-methylhexanedioic acid	9.33	C_10_H_16_O_6_	231.0866		231/213/169/125/157/187/143	−	−	−	−	++	[28]
6	Protocatechuic acid	11.39	C_7_H_6_O_4_	153.0179		109/153/110	−	−	+	−	−	[29]
7	Vicenin-1	14.72	C_26_H_28_O_14_	563.1413		563/353/383/297/473/443/297/325/503/545	−	−	−	+	+	[30]
8	Homoorientin	15.15	C_21_H_20_O_11_	447.0935		429/357/327	−	−	+	−	+	Standard
9	Orientin	15.77	C_21_H_20_O_11_	447.0937		369/357/327/299	−	−	+	+	−	Standard
10	Agnuside	16.04	C_22_H_26_O_11_	465.1404		303/285/281/165/137/121/93/89	−	−	+	−	−	[31]
11	Vitexin	17.72	C_21_H_20_O_10_	431.0986		311/431/283/341/323	−	−	−	+	−	Standard
12	20-Hydroxyecdysone	19.15	C_27_H_44_O_7_	525.3073	481.3152	445/371/165/427/125/69/481	−	−	++	+++	++	[32]
13	Luteolin-7-O-glucuronide	19.57	C_21_H_18_O_12_	461.0734		285	−	−	+	+	+	[33]
14	3,4-dicaffeoylquinic acid	22.22	C_25_H_24_O_12_	515.1201		353/191/179	−	−	+++	−	−	Standard
15	Luteolin-4′-O-glucoside	23.84	C_21_H_20_O_11_	447.0939		285/447	−	−	−	+	−	Standard
16	Isovitexirone	24.18	C_27_H_42_O_7_	523.2917	479.2998	425/443/373/123/145/219/303/407/461/479	−	−	+	++	−	[33]
17	Vitexirone	26.57	C_27_H_42_O_7_	523.2917	479.2998	69/443/425/299/109/407/479/461/281/311	−	−	+	++	−	[34]
18	Pterosterone	27.84	C_27_H_44_O_7_	525.3074	481.3152	445/427/371/125/69	−	−	+	+	−	[35]
19	Ajugasterone C	32.70	C_27_H_44_O_7_	525.3073	481.3152	427/445/81/409/299/311/481/463/189	−	−	++	++	−	[36]
20	Luteolin	38.57	C_15_H_10_O_6_	285.0405		285/286/133/151/175	+	−	+++	−	−	Standard
21	Rosmarinic acid	40.98	C_18_H_16_O_8_	359.0777		344/329/359/314/286	+	+	++	−	−	Standard
22	3,7-Dimethylquercetin	40.99	C_17_H_14_O_7_	329.0664		314/299/271/243	++	++	−	−	−	Standard
23	Eupatorin	41.82	C_18_H_16_O_7_	343.0824		328/298/270	+	++	−	−	−	[37]
24	Octadecenedioic acid	42.26	C_18_H_32_O_4_	311.2232		293	+++	+	−	−	−	[38]

−: Absence; +: Low content; ++: high content; +++: very high content.

**Table 2:** 1H and 13C data of 20-hydroxyecdysone, ajugasterone C, vitexirone and pterosterone.

	*20-Hydroxyecdysone*		*Ajugasterone C*		*Vitexirone*		*Pterosterone*	
**Position**	**δ_C_ type**	**δ_H_ (*J* in Hz)**	**δ_C_ type**	**δ_H_ (*J* in Hz)**	**δ_C_ type**	**δ_H_ (*J* in Hz)**	**δ_C_ type**	**δ_H_ (*J* in Hz)**
1a	37.5 CH_2_	1.78 m	39 CH_2_	2.59 dd (13.0, 4.0)	38.8 CH_2_	2.60 dd (13.0, 4.0)	35.7 CH_2_	1.74 m
1b		1.43 m		1.38 m		1.76 m		1.35 m
2	68.8 CH	3.83 ddd (11.5, 4.0, 3.5)	68.9 CH	4.02 td (12.0, 4.0)	68.4 CH	4.00 td (12.0, 5.0, 4.0)	68.7 CH	3.83 m
3	68.6 CH	3.96 m	68.6 CH	3.96 m	68.7 CH_2_	3.95 m d (8.4)	68.5 CH	3.95 m
4a	32.8 CH_2_	1.72 m	33.3 CH_2_	1.79 m	33.0 CH_2_	1.71 m	32.8 CH_2_	1.72 m
4b		1.69 m		1.68 m		1.65 m		1.72 m
5	51.7 CH	2.38 dd (12.5, 4.5)	52.6 CH	2.34 dd (12.5, 4.0)	52.2 CH	2.34 dd (13.0, 4.0)	51.7 CH	2.37 dd (12.5, 4.5)
6	206.5 C		206.7 C		206.2 C	7.26, s	206.5 C	
7	122.1 CH	5.81 d (2.5)	122.7 CH	5.79 d (2.5)	122.4 CH	5.80 d (2.5)	122.0 CH	5.80 d (2.5)
8	168.2 C		165.7 C		165.4 C		167.9 C	
9	35.0 CH	3.16 t (8.5)	42.8 CH	3.15 dd (8.5, 2.5)	42.6, CH_2_	3.15, dd (8,5, 2.5)	35.1 CH	3.14 m
10	39.2 C		39.8 C		39.5 C		39.2 C	
11a	21.7 CH_2_	1.81 m	69.4 CH	4.09 m	69.4 CH	4.10m	21.4 CH_2_	1.74 m
11b		1.70 m						1.70 m
12a	32.5 CH_2_	2.14 td (13.0, 4.5)	43.7 CH_2_	2.21 m	43.5 CH_2_	2.26 m	32.4 CH_2_	2.11 td (13.0, 4.5)
12b		1.88 m		2.17 m		2.16 m		1.88 m
13	48.7 C		48.7 C		48.1 C		47.8 C	
14	85.3 C		84.8 C		84.3 C		85.2 C	
15a	31.8 CH_2_	1.96 m	31.8 CH_2_	1.97 m	31.4 CH_2_	2.32 m	31.7 CH_2_	1.96 m
15b		1.60 m		1.59 m		1.58 m		1.60 m
16a	21.4 CH_2_	1.98 m	21.4 CH_2_ C	1.99 m	21.3 CH_2_ C	1.97 m	21.5 CH_2_	2.00 m
16b		1.72 m		1.71 m		1.77 m		1.69 m
17	50.5 CH	2.39 dd (8.0, 4.5)	50.2 CH	2.41 m	50.2 CH	2.39 dd (9.0, 4.0)	50.5 CH	2.32 m
18	18.0 CH_3_	0.89 s	18.8 CH_3_	0.87 s	18.6 CH_3_	0.86 s	17.0 CH_3_	0.90 s
19	24.3 CH_3_	0.96 s	24.7 CH_3_	1.05 s	24.3 CH_3_	1.06 s	24.4 CH_3_	0.98 s
20	77.8 C		77.7 C		77.2 C		77.8 C	
21	21.1 CH_3_	1.20 s	20.7 CH_3_	1.19 s	20.7 CH_3_	1.21 s	20.9 CH_3_	1.21 s
22	78.4 CH	3.33 m	77.8 CH	3.34 m	77.7 CH	3.34 dd (10.0, 2.5)	77.5 CH	3.56 m
23a	27.3 CH_2_	1.65 m	30.4 CH_2_	1.57 m	31.6 CH_2_	2.28 m	35.7 CH_2_	1.58 m
23b		1.28 m		1.22 m		1.97 m		1.25 m
24a	41.9 CH_2_	1.79 m	37.7 CH_2_	1.47 m	122.9 CH	5,26 t (7)	77.6 CH	3.60 m
24b		1.42 m		1.23 m				
25	71.3 C		29.2 CH	1.58 m			32.2 CH	1.60 m
26	28.9 CH_3_	1.18 s	22.6 CH_3_	0.91 d (6.5)	25.7 CH_3_	1.71 s	16.9 CH_3_	0.93 d (6.5)
*27*	*29.7 CH_3_*	*1.19 s*	*23.5 CH_3_*	*1.19 d (6.5)*	*17.8 CH_3_*	*1.65 s*	*19.3* *CH_3_*	*0.97 d (6.5)*

## Data Availability

The original contributions presented in this study are included in the article. Further inquiries can be directed to the corresponding author(s).
